# How Can We Understand the Genomic Basis of Nematode Parasitism?

**DOI:** 10.1016/j.pt.2017.01.014

**Published:** 2017-06

**Authors:** Mark Viney

**Affiliations:** 1 School of Biological Sciences, University of Bristol, Tyndall Avenue, Bristol, BS8 1TQ, UK

## Abstract

Nematodes are very common animals and they have repeatedly evolved parasitic lifestyles during their evolutionary history. Recently, the genomes of many nematodes, especially parasitic species, have been determined, potentially giving an insight into the genetic and genomic basis of nematodes’ parasitism. But, to achieve this, phylogenetically appropriate comparisons of genomes of free-living and parasitic species are needed. Achieving this has often been hampered by the relative lack of information about key free-living species. While such comparative approaches will eventually succeed, I suggest that a synthetic biology approach – moving free-living nematodes towards a parasitic lifestyle – will be our ultimate test of truly understanding the genetic and genomic basis of nematode parasitism.

## Trends

Genomic analyses of nematodes has the potential to give critical insights into the genetic and genomic basis of nematode parasitism.

Comparisons of the genomes of free-living and parasitic nematodes need to be phylogenetically appropriate, to properly understand the genomic basis of nematode parasitism.

We lack information about free-living species that are close relatives of parasitic nematodes of interest.

Our immense accumulated understanding of the biology of the model *Caenorhabditis elegans* might lead us astray, because we can forget that it is just one, perfectly average, nematode.

Synthetic biology approaches may give us the opportunity ultimately to test our understanding of the basis of nematode parasitism.

## Parasitism Is Common

Being a parasite is a very common lifestyle; in fact, it’s been convincingly argued that it is the most common lifestyle 1, 2. There are numerous definitions of what parasitism is, but here it is taken to mean an organism living in or on another organism, from which the parasite obtains benefit to the detriment of the host. Phylogenetic analysis of the occurrence of parasitism among animals shows that parasitism has arisen at least 200 times in 15 phyla [3]. While parasitism is a common lifestyle among animals, some groups of invertebrates – for example the bryozoa and echinodermata – are notable for never having evolved parasitism [3]. Notwithstanding, the commonness of parasitism makes good sense: hosts are rich patches of resource ready to be exploited, and evolution has acted to do this.

Nematodes are the most abundant and speciose group of living animals and many species have a parasitic lifestyle. Parasitic nematodes are important parasites of humans and domestic animals [4]. More broadly, nematode parasitism is ubiquitous: all species of animals and plants are parasitized by nematodes [5]. Nematodes parasitize many types of organism, though studies of parasites of animals predominate, and this bias will be maintained here.

## Why Have Nematodes Commonly Evolved Parasitism?

Phylogenetic analyses show that nematodes have evolved to be parasites on up to 18 separate occasions in their evolutionary history 3, 5, 6, 7. This provokes the question, what is it about nematodes that has facilitated their evolution of parasitism? Is it that for nematodes there are just a few simple evolutionary steps from having a free-living lifestyle to becoming a parasite (whereas for other taxa, there are more such evolutionary steps, and that are more tortuous, and so less likely to occur)? Or, is it actually that free-living nematodes’ biology and ecology means that they are commonly living with other animals and plants, so that there are often opportunities to evolve more interdependent relationships with other taxa, leading in time to parasitism? A third possibility is simply that because nematodes are so abundant and speciose, that natural selection has had more individuals on which to work and in more settings (compared with taxa that are less abundant and less speciose), so that of course nematodes will have repeatedly evolved parasitism. Of course, these possible explanations are not mutually exclusive.

A common narrative for the evolution of nematode parasitism is that a free-living ancestral species began to live in close association with another animal (or plant). Then, over time, this relationship became ever more intimate. As this happened, the nematode began to specialise at exploiting its new niche, with the result that eventually the nematode became dependent on the other species, such that the nematode was now an obligate parasite and unable to live without, what had now become, its host.

For nematodes, all the elements necessary for this scenario to occur are in place. Perhaps of particular importance is that free-living nematodes do live in close association with other organisms. For example, the well studied free-living nematode *Caenorhabditis elegans* (of which more later) lives in rotting vegetation, but it is also frequently found associated with other invertebrate animals, particularly molluscs (slugs and snails) and arthropods 8, 9, 10, 11, 12, 13. *C. elegans* has even been shown experimentally to survive brief passage through slug guts, showing its potential for parasitism [13]. However, when *C. elegans* is found on molluscs and insects it isn’t clear whether the worms are only occasionally living on them, perhaps just accidently ending-up on them, or whether the worms are actively seeking out these animals as desirable habitats. This also points to types of association between species – mutualism, commensalism, phoresy, etc. – beyond parasitism in which nematodes likely engage.

Study of another nematode, *Pristionchus pacificus*, is also instructive here. This species was adopted as a new model free-living nematode system to complement *C. elegans*. Initially, *P. pacificus* was commonly found free in soil and could be grown, like *C. elegans*, on a simple bacterial food source on agar plates. Only latterly was it discovered that in nature *P. pacificus* actually lives in close association with species of beetles [14]. Moreover, *P. pacificus*’s principal life-history is to live in association with these beetles; being separated from the beetles and living in soil is likely rare or aberrant. More generally, the biology of these two free-living species (or perhaps that should be *putatively* free-living species) shows that associations between free-living nematodes and other animals might be common, and that these are, perhaps, the ideal settings in which parasitism can evolve.

One key aspect of nematode biology that might be very important in their evolution of parasitism is that nematodes are moulting animals. The standard nematode life cycle consists of an adult stage with four larval stages occurring between adult generations. This basic life cycle plan has been modified in many species, but the four larval-stage pattern is still always apparent in all nematode life cycles. When nematodes moult they renew their surface cuticle, in large part to allow them to grow [15]. But, beyond this, as they moult they are also able to reset their biology and physiology. For example, nematode surface cuticles are largely composed of collagen. *C. elegans* has some 170 collagen coding gene family members, and suites of different collagen coding genes are used by each different larval and adult stage [16]. This means that *C. elegans* can alter the structure of its cuticle each time it moults [16]. This then is one example of the more general phenomena whereby nematodes can alter their biology when they moult. This might be particularly important as nematodes evolved parasitic lifestyles, because moulting (and other associated changes in biology that can occur at a moult) can allow certain life-cycle stages to have a physiology appropriate to living inside a host, while other life-cycle stages can have a biology and physiology that is appropriate for a free-living lifestyle, with these transitions able to occur at moults [17]. In parasitic nematode life cycles, transitions from free-living transmission stages into a within-host parasitic stage (for example, infective larvae of hookworms and *Strongyloides* moving into definitive hosts), or transitions between different host species (for example, infective larvae of filarial nematodes moving from an arthropod vector to a definitive host), are usually coincident with moulting 5, 18.

Evolutionary change commonly occurs by organisms co-opting existing traits to a new purpose. Therefore thinking about the evolution of nematode parasitism, we need to ask what traits of free-living nematode species have been co-opted in the evolution of parasitism, and how have these co-opted traits changed with the evolution of parasitism? Similarly, to understand the genetic basis of nematode parasitism, for nematode genomes what genes and gene families have been co-opted and changed in the evolution of parasitism? But, we must be cautious in this endeavour because, as evolutionary developmental biology has shown, phenotypic similarity can belie extensive genetic differences 19, 20.

## Using Phylogenetic Approaches to Understand Nematode Parasitism

In the same way that phylogenetic analyses have been used to understand the evolution of parasitism among animals and among nematodes, phylogenomic comparisons can be used to understand how nematode genomes have changed as parasitism evolved, and so to suggest the genetic and genomic basis of nematode parasitism. Crucial to the success of these analyses is making the correct comparisons. For nematodes, because parasitism has evolved multiple times, informative genomic comparisons between free-living and parasitic species are only possible when the species being compared are from within the same nematode clade or subclade. This is because such comparisons involve the point at which the relevant evolutionary transition from a free-living to a parasitic lifestyle occurred.

**Figure 1:**
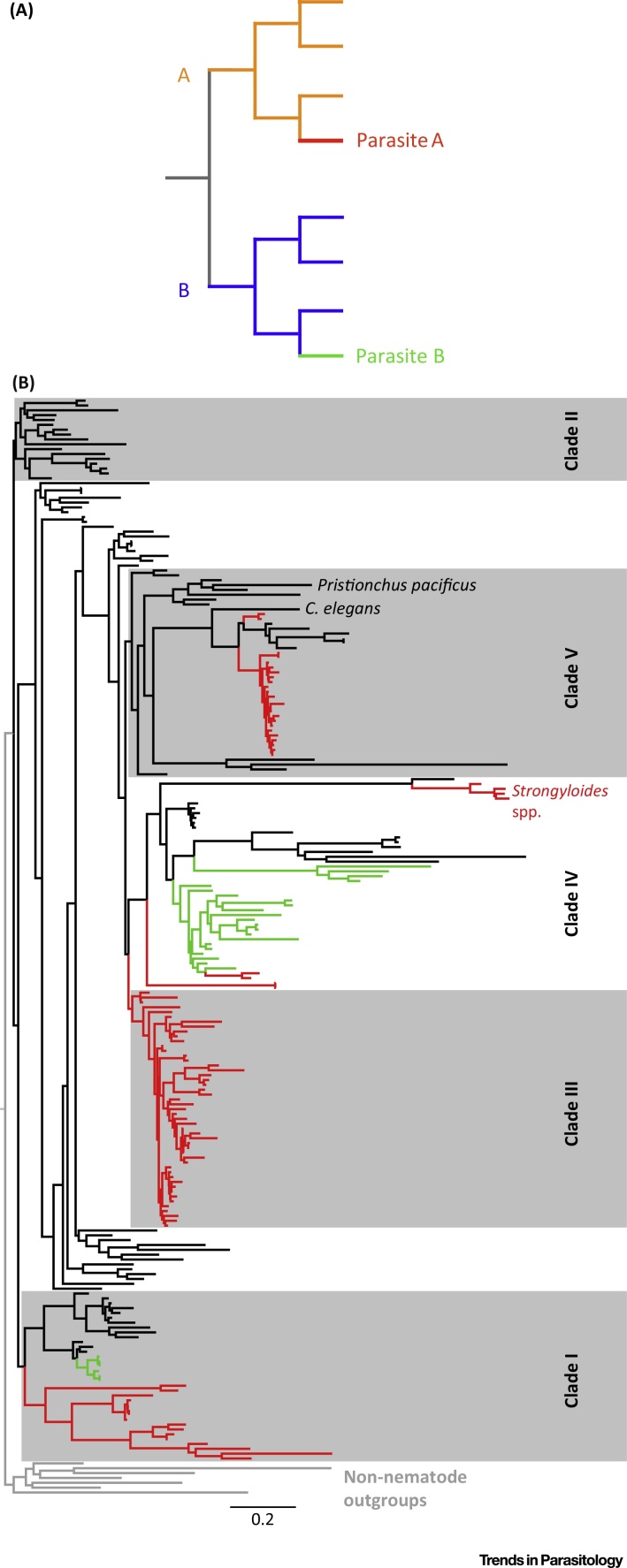
Phylogenetic Comparisons to Understand Nematode Parasitism. (A) A hypothetical phylogeny of two nematodes clades, A and B, where the ancestral species had a free-living lifestyle. Within each clade parasitism evolves giving parasite species A in clade A and parasite species B in clade B. Comparisons of parasite species A with free-living species within its own clade is informative to understand the evolution of parasitism within that clade. Comparisons across clades can confound the clade-specific evolutionary history with the species’ lifestyle. (B) A phylogeny of 226 nematode species based on an alignment of 18S rRNA from release 128 of the SILVA rRNA database [35], where animal parasites are in red, plant parasites are in green, and free-living species are in black, showing the principal nematode clades (I–V) after [6], showing *Pristionchus pacificus, C. elegans* and *Strongyloides* spp., which are mentioned in the text. Scale is expected substitutions per nucleotide site.

Consider the hypothetical nematode phylogeny of Figure 1, where there are two nematode clades, each of which has independently evolved parasitism. Comparison of parasite species A with free-living species within clade A compares taxa with the same evolutionary history, so that differences between these species can justifiably be used to infer what underlies their different lifestyles. In contrast, comparing parasite species B with free-living species in clade A compares taxa with different evolutionary histories, so differences between them are both due to the different evolutionary histories of their clades, as well as their differences in lifestyle. In this comparison, one cannot therefore be certain what genomic changes are to do with parasitic or free-living lifestyles, and what are to do with differences between clades. Put simply, this would be a confounded analysis. The actual phylogeny of nematodes shows that there is additional complexity (Figure 1). In some nematode clades (e.g., clade III) there appear to be no close free-living relatives to the parasitic species, though for other taxa there are related free-living and parasitic species (e.g., clade V).

Despite the evolutionary convergence of many parasitism traits among nematodes, this does not necessarily mean that there is underlying genomic convergence 7, 21. One example will serve to demonstrate this. The arrested third larval stage of *C. elegans* (and many other free-living nematodes), known as dauer larvae, are a stage that is commonly thought to be analogous to the infective larvae of many parasitic nematodes. Genetic and genomic analyses have shown that the genes controlling the development of the *C. elegans* larval arrest are not universally present among other parasitic nematode species [21]. Moreover, even when the same genes are present in free-living and parasitic nematode species, they can be co-opted into different functions 22, 23, 24. So the phenotypic analogy of free-living nematode arrested larvae and parasitic nematode infective larvae is probably not underpinned by an analogous molecular control. This does not necessarily mean that the phenotypic analogy is wrong, but simply that genetic control of phenotypes can evolve without apparently changing the phenotype [19]. This again argues for phylogenetically appropriate comparisons between free-living and parasitic taxa when trying to understand the evolution of parasitism.

**Figure 2:**
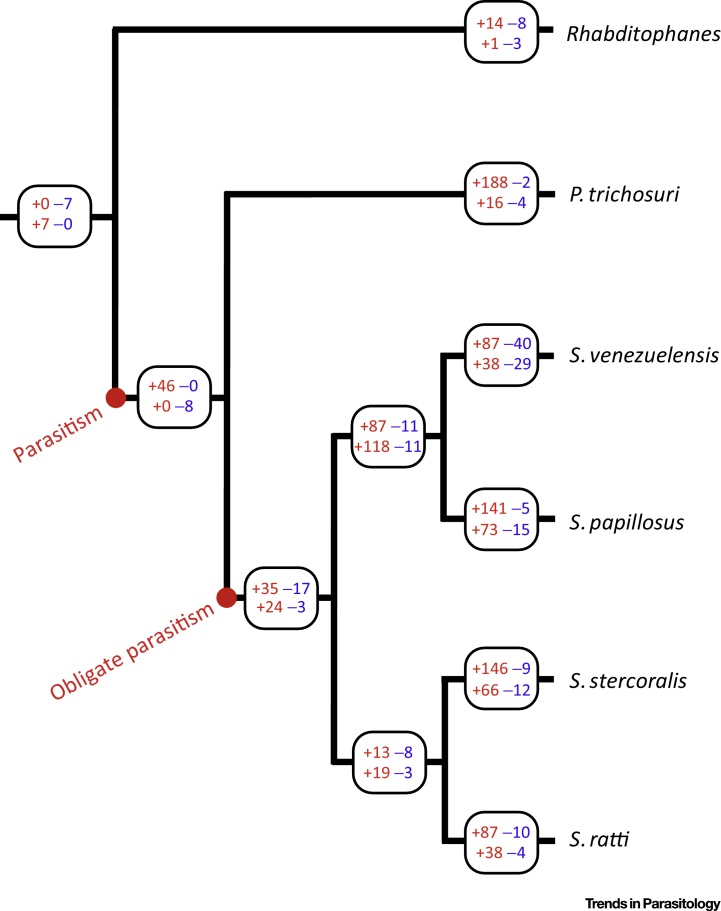
The Phylogeny of *Strongyloides* and Its Relatives and the Evolution of Parasitism. A phylogeny of four species of *Strongyloides, Parastrongyloides trichosuri*, and *Rhabditophanes* sp. *Rhabditophanes* is a free-living species. *Parastrongyloides* can have multiple free-living adult generations, but can also be parasitic, making it a facultative parasite. *Strongyloides* is an obligate parasitic species because, even though it has a free-living adult generation (as does *Parastrongyloides*), *Strongyloides*’ life cycle requires a parasitic adult generation every generation. Where parasitism and obligate parasitism are inferred to have arisen is shown. The boxes show the number of astacin metallopedtidase (top) and SCP/TAPS protein (bottom) coding genes that have been acquired (+, in red) and lost (–, in blue) across the phylogeny. The tree branch lengths do not show the relative distance among the taxa. Data from [25].

Our own work with *Strongyloides* has used this phylogenomic approach to understand the basis of parasitism within a clade of nematodes [25]. Here we were able to compare the genomes of four species of *Strongyloides*, with its close relative, the facultative parasite *Parastrongyloides*, and with the closely related free-living species *Rhabditophanes* (Figure 2). The phylogenetic relatedness of these taxa, and the free-living vs. parasitic lifestyle differences among them, furnished the opportunity to work out what genes and gene families changed, and how they changed, across the phylogeny. In these analyses, the genome state at each node of the phylogeny – each of which represents hypothetical ancestors to the extant species – was calculated, which gives a clear view of the changes in genes and gene families that accompanied *Strongyloides*’ evolution of parasitism.

Overall, this showed that the parasitic species (*Strongyloides* and *Parastrongyloides*) had acquired new gene families (compared with ancestors) and that on the evolutionary branches to each of the five parasitic species, this acquisition of genes was concentrated in a fairly limited number of gene families (Figure 2). For *Strongyloides* and *Parastrongyloides*, gene families coding for astacin metallopeptidases and SCP/TAPS proteins particularly expanded with the evolution of parasitism (Figure 2). Beyond these, other gene families that became particularly comparatively enlarged as parasitism evolved include those coding for acetylcholinesterases and prolyl endopeptidases [25]. Some of these gene families have been suggested to be associated with the parasitic lifestyle of other parasitic nematodes – particularly various protease-coding genes [7] as well as SCP/TAPS protein coding genes suggesting that these proteins may play a fundamental role in nematode parasitism more generally [26]. Proteases may have a range of roles, including being used by the parasite to move through host tissue, and to provide food to the parasite [26]. For example, *Strongyloides* parasitic adults actually live in tunnels in the mucosa of the host's gut, and secreted proteases are probably used to excavate these tunnels [27]. Parasite proteases are also thought to be used against molecules of the host immune response, so acting as an immune defence mechanism. While identifying the gene families that might be involved in the parasitic lifestyle is important, finding out what role they play in this lifestyle is considerably harder 26, 28.

## Better Phylogenies

Crucial to the success of using phylogenomic approaches to understand how nematode genomes have evolved to drive parasitic lifestyles, is making appropriate and informative comparisons of free-living and parasitic taxa. Nematode parasite species are well known and studied, and there are a plethora of parasitic nematode genomes now available (e.g., http://parasite.wormbase.org). Arguably, what is lacking is knowledge (let alone genomic information) about species of free-living nematodes that are close relatives of parasites of interest 5, 29. Therefore, a better understanding of the genomic basis of nematode parasitism will come from a concerted effort at identifying and genome sequencing free-living nematodes that are close relatives to parasites of interest. The existing phylogeny (Figure 1) shows where such effort could usefully be directed. For some nematode clades, the way to achieve this is to work to discover hitherto unknown free-living species. For some nematode clades closely related free-living species may not exist, and in these situations then carefully phylogenetically judged next-nearest neighbours could be used, as too can any species that have reverted to a free-living lifestyle [30].

**Is *C. elegans* a Problem?**

The ‘archetypal’ free-living nematode is *C. elegans*. Its genome was the first metazoan genome ever sequenced and now there are genome sequences of many other *Caenorhabditis* spp. (http://wormbase.org). But beyond this genus (with the exception of *Pristionchus* spp., though this is in the same nematode clade as *C. elegans*), we know rather little of free-living nematodes. In parasitic nematode biology there is always the obvious temptation to compare parasitic nematodes and their genomes to *C. elegans*, but this can lead to false conclusions, because nematode clade and lifestyle can be confounded (Figure 1).

The huge, concentrated and long-term research effort expended on understanding *C. elegans* has been of great importance in nematode biology. But, has *C. elegans* now perhaps become a hindrance in understanding nematodes more widely? Why? Because it can be a struggle to think that nematodes can be different from *C. elegans.* It is clear, and it continues to get clearer still, that *C. elegans* is just one nematode and that most aspects of its biology differ from that of other *Caenorhabditis* spp. The more *C. elegans* is studied, the more its uniqueness becomes clear. But this is a falsehood of observation – if anything is studied in ever greater detail, then it necessarily becomes more different, and eventually unique, compared with everything else. So while *C. elegans* might do something one way, other *Caenorhabditis* spp. will do the same thing differently, and other free-living nematodes will use yet other ways to achieve the same end. If Sydney Brenner had chosen a nematode other than *C. elegans* [31] our starting point for understanding nematodes would be very different. While what we know about *C. elegans* is very interesting, it’s important that we remember that it’s only one, not very special, worm – or at least that it is a worm that is only as special as all the other, lesser studied ones.

## Using Synthetic Biology to Understand Nematode Parasitism

Phylogenomic approaches are powerful ways to interrogate nematode genomes to understand the genetic basis of parasitic lifestyles. But, necessarily, these approaches make predictions which are not easy to test.

In model organism biology the classic way to discover the role of a gene is to study the phenotype of the organism where the gene of interest does not function. The gold-standard approach to proving that the gene in question actually underlies the phenotype is to rescue the mutant phenotype by transforming the mutant with the wild-type allele of the gene. This is a relatively straightforward approach (once one has the genetic tools [32]), especially for traits where a small number of genes have large phenotypic effects. This approach is the backbone of the success of the *C. elegans* field.

But, using this approach to study the genetics of the evolution of nematode parasitism would seem unlikely to be successful, possibly pointless (and likely unfundable) for two interrelated reasons. Firstly, for macro evolutionary changes, such as a transition from a free-living to a parasitic lifestyle, there will be a whole series of coordinated changes in life-history biology, physiology, and structure, all of which will contribute to the trait of ‘parasitism’. Put another away, there are likely to be many genes each of small effect – so that it’s most unlikely that a small number of genes will in any sense define parasitism. Secondly, even if a few identifiable genes did define parasitism, knock-out of those genes would likely be a lethal phenotype – the parasite would no longer be a parasite. But, lethal phenotypes are hard to work with and unlikely to be informative when trying to understand parasitism. Furthermore, for all organisms there are many genes that when knocked-out result in a lethal phenotype, and so our challenge would then become to identify which of these lethal-when-knocked-out genes are ones key of parasitism, as opposed to key to life itself.

Instead, I suggest that a better way to experimentally test our understanding of parasitic nematodes would be to engineer a free-living nematode towards parasitism. The evolution of a fully-parasitic species is a macro evolutionary event, so engineering a fully-fledged parasitic nematode may be a step too far. So, instead, can we engineer free-living nematodes for the initial micro evolutionary events that put them on the path to parasitism, so that such an engineered free-living nematode would acquire some traits related to parasitism?

*C. elegans* is the obvious test vehicle, not only because it has robust genetic tools available in abundance, but also because its natural ecology includes living in close association with other animals. Many genes of parasites have been expressed in *C. elegans*, but not with the intention of driving parasitism traits in this species. Rather, the question I am posing is, can we engineer *C. elegans* to acquire parasitism-related traits? We would not be seeking to convert *C. elegans* into a fully functioning, obligate parasitic nematode, but rather to give it some parasitism-related traits.

How might such an engineering approach be used? Suppose that from phylogenetically informed analyses we conclude that parasitic nematodes (ideally of nematode clade V, to which *C. elegans* belongs [5]) have evolved an expanded gene family of a family of proteases (see above). Our inference from this phylogenetic observation is that the possession of these gene families in some way underlies these species' parasitic lifestyle. So let’s test it, by engineering *C. elegans* to contain and express these expanded gene families. Our prediction is that this engineered *C. elegans* will now have acquired parasitism-associated traits. We can test this prediction too. Because wild-type *C. elegans* can live on molluscs and insects, we can compare wild-type and parasitism-engineered *C. elegans* on these animals. Our prediction of this experiment is that the parasitism-engineered worms will live better, longer, with a greater fecundity, and more intimately with the host, compared with the wild-type *C. elegans*. It is of course critical that we are thoughtful in these experiments. For example, one could engineer *C. elegans* to express a bacterial-derived vertebrate toxin, administer these worms to animals, and then (rashly) conclude that one had engineered a highly virulent nematode parasite. But, this is to misunderstand what is proposed, which is that phylogenetically appropriate analysis of parasite genomes must direct the genes that are targeted.

Synthetic Biology – defined in many ways, but here taken to mean the rational and predictable design and engineering of biological systems – is all the vogue just now. We could use synthetic biology’s approach to iteratively, rationally, and predictably engineer a nematode proto-parasite. In this we can test our growing understanding of the genomic basis of nematode parasitism to ask if we can synthetically engineer a nematode with parasitism traits. The key reason to do this is to rigorously test our understanding of the genetic basis of nematode parasitism, but there might be other uses too. For example, a synthetic parasite could be used for practical purposes, aimed at improving human and animal health: (i) as an antinematode drug discovery model, (ii) as an antinematode vaccine-delivery vehicle, especially because the site and manner of antigen presentation profoundly affects vaccination efficacy (and *C. elegans* is already known to induce a typical antihelminth Th2 immune response [33]), (iii) as an immunomodulatory therapy by harnessing a synthetic parasitic nematode’s ability to immunomodulate hosts [34].

Experimental evolution of a free-living nematode (again, *C. elegans* being the obvious choice) is a complementary approach that could be used to study the evolution of parasitism. Here, using the established association between *C. elegans* and molluscs and insects, can *C. elegans* be selected to live for longer, with higher fecundity, and more intimately, on such hosts? As noted above, such an experimental evolution approach would not realistically seek to achieve the macro evolutionary transition to complete parasitism, but would instead seek micro evolutionary changes associated with a parasitic lifestyle. Genomic analyses of such experimentally evolved populations could then be used to understand what genetic changes underlie the parasitism-associated phenotypic changes.

## Concluding Remarks

Nematode parasites are tremendously common, and cause immense harm to humans and other animals. How these organisms have evolved to be parasites is a question that has long puzzled zoologists (see Outstanding Questions). Now, by using carefully chosen, appropriate phylogenomic analyses we should finally be able to understand how nematodes have evolved parasitism. Answering this will be a major advance, but doing so will also help in efforts to control these parasites and the harm that they cause. Arguably, we need a better understanding of free-living nematode species to properly understand how nematode parasitism evolved. Looking forward, we should be able to test our understanding of nematodes’ evolution of parasitism by using synthetic biology approaches to convert artificially a free-living nematode towards a parasitic lifestyle. When we do this, we will know that we have truly understood the genetic and genomic basis of nematode parasitism.

Outstanding Questions How do free-living nematodes evolve a parasitic lifestyle, and what are the genetic and genomic changes underlying this transition?

For which parasitic nematodes do we need to find phylogenetically close free-living relatives to properly understand parasites’ genomic adaptations to parasitism?

Recognizing that phenotypic similarity often masks an underlying diversity of mechanisms of molecular control, across different clades of nematodes, what is common and what is clade-specific in their evolution of parasitism?

How can we use a basic understanding of how nematodes evolved parasitism to improve existing, and develop new, ways to control them?

What is the role of free-living nematodes’ arrested dauer larvae in the evolution of nematode parasitism?

Is there such a thing as a truly free-living nematode?
